# Enabling deeper learning on big data for materials informatics applications

**DOI:** 10.1038/s41598-021-83193-1

**Published:** 2021-02-19

**Authors:** Dipendra Jha, Vishu Gupta, Logan Ward, Zijiang Yang, Christopher Wolverton, Ian Foster, Wei-keng Liao, Alok Choudhary, Ankit Agrawal

**Affiliations:** 1 Department of Electrical and Computer Engineering, Northwestern University, Evanston, USA; 2 Computation Institute, University of Chicago, Chicago, USA; 3 Data Science and Learning Division, Argonne National Laboratory, Lemont, USA; 4 Department of Materials Science and Engineering, Northwestern University, Evanston, USA

**Keywords:** Computational methods, Materials science

## Abstract

The application of machine learning (ML) techniques in materials science has attracted significant attention in recent years, due to their impressive ability to efficiently extract data-driven linkages from various input materials representations to their output properties. While the application of traditional ML techniques has become quite ubiquitous, there have been limited applications of more advanced deep learning (DL) techniques, primarily because big materials datasets are relatively rare. Given the demonstrated potential and advantages of DL and the increasing availability of big materials datasets, it is attractive to go for deeper neural networks in a bid to boost model performance, but in reality, it leads to performance degradation due to the vanishing gradient problem. In this paper, we address the question of how to enable deeper learning for cases where big materials data is available. Here, we present a general deep learning framework based on Individual Residual learning (IRNet) composed of very deep neural networks that can work with any vector-based materials representation as input to build accurate property prediction models. We find that the proposed IRNet models can not only successfully alleviate the vanishing gradient problem and enable deeper learning, but also lead to significantly (up to 47%) better model accuracy as compared to plain deep neural networks and traditional ML techniques for a given input materials representation in the presence of big data.

## Introduction

The collection of large scale datasets through experiments and first-principles calculations such as high throughput density functional theory (DFT) computations^1–7^ along with the emergence of integrated data collections and registries^8,9^ have spurred the interest of materials scientists in applying machine learning (ML) models to understand materials and predict their properties^10–19^. Due to their impressive ability to efficiently extract data-driven linkages between various materials representations (composition- and/or structure-dependent) in the model input and their properties at the model output, the application of machine learning (ML) techniques in materials science has attracted significant attention throughout the materials science research community. Such interests have been supported by government initiatives such as the Materials Genome Initiative (MGI)^20^, leading to the novel data-driven paradigm of materials informatics^15,21–25^.

While the application of traditional ML techniques such as Random Forest, Support Vector Machine and Kernel Regression, has become ubiquitous in materials science^10–19^, the applications of more advanced deep learning (DL) are still limited^26–34^. SchNet^26^ used continuous filter convolutional layers to model quantum interactions in molecules for the total energy and interatomic forces that follows fundamental quantum chemical principles. SchNet is extended with an edge update network to allow for neural message passing between atoms for better predictions of properties of molecules and materails in^27^. Zhou et al.^35^ used a fully connected network with single hidden layer to predict formation energy from high-dimensional vectors learned using Atom2Vec. ElemNet^28^ leveraged a deep neural network to automatically capture the essential chemistry between elements from elemental fractions to predict the formation enthalpy of materials without using any domain knowledge based feature engineering. ElemNet is used for transfer learning from large DFT dataset to experimental dataset for more accurate prediction of formation enthalpy closer to true experimental observations^32^. Crystal graph convolution neural networks are used to directly learn material properties from the connection of atoms in the crystal, providing a universal and interpretable representation of crystalline materials^29^. Park et al. improved the crystal graph convolutional neural networks by incorporating information of the Voronoi tessellated crystal structure, explicit 3-body correlations of neighboring constituent atoms, and an optimized chemical representation of interatomic bonds in the crystal graph, for accelerated materials discovery. Recently, Goodall and Lee^33^ developed a machine learning approach that takes only the stoichiometry as input and automatically learns appropriate and systematically improvable materials descriptors from data using a message-passing neural network by reformulating the stoichiometric formula of a material as a dense weighted graph between its elements. Chen et al. developed a universal MatErials Graph Network (MEGNet) model for materials property prediction of molecules and cyrstals^36^. All these DL works generally focus on learning either the material embeddings or the atomic interactions using graph networks from the crystal structure^26,29,30,36^. Although deeper architectures are believed to lead to better performance when big data is available, current neural networks used in materials science applications do not leverage deeper architectures.

Recently, there has been a drastic shift towards leveraging deeper neural network architectures in computer science fields such as computer vision and natural language processing^37–45^. These networks are composed of up to hundreds of layers/blocks, which enable them to capture the high level abstract features from the big input training datasets^46–50^. Such deep neural networks have been possible because of several attempts^38,39,51,52^ to address the performance degradation issue due to vanishing and/or exploding gradient problem. Generally model parameters are initialized to small magnitudes in the range of [0,1] during training, and the normally used activation functions have gradients in the range of [− 1, 1]. During backpropagation, the gradients are computed at each layer to update the model parameters by applying the chain rule of partial derivatives with respect to the cost function from the output layer^53^. This successive multiplication of the gradients with numbers of small magnitude can lead to an exponential decrease in the magnitude of gradients (which are responsible for parameter updates), as they flow from the output layer to the initial layers during backpropagation, which effectively halts further training of the network. This is known as the vanishing gradient problem in deep neural networks. Similarly, the exploding gradient problem can happen when the computed error at the output layer has an extremely large magnitude, possibly due to overflowing in some model parameters during forward propagation; this can lead to huge updates in model parameters during backpropagation, rendering them inappropriate for convergence with further training.

Although materials datasets are typically not as big as the image and text datasets used in computer science applications, they can still contain hundreds of thousands of samples at present and are regularly growing in size^1–7^. Given the demonstrated potential and advantages of leveraging such deeper neural network architectures in computer science, it is attractive to go for deeper neural network architectures in a bid to boost model performance in the presence of big materials datasets. Hence, rather than focusing on designing a neural network to learn another type of materials representation or embedding as in recent works^26,29,30,33,36^, here, we focus on addressing the general issue of how to develop deeper neural network architectures for more accurate and robust prediction performance in the presence of big data for a given material representation.

We present a general deep learning framework composed of deep neural networks (from 10-layer up to 48-layer) based on Individual Residual learning (IRNet) that can learn to predict any material property from any vector-based given material representation (composition- and/or structure derived attributes). Since the model input contains a vector of independent features, the model architectures are composed of fully connected layers. Fully connected layers contain huge number of parameters proportional to the product of input and output dimensions. There have been several approaches to deal with the performance degradation issue due to vanishing and/or exploding gradient problem. To address this issue for deep neural networks with fully connected layers, we present a novel approach of residual learning based on He et al.^38^; other approaches^39,52^ will result in a tremendous increase in the number of model parameters, which could lead to GPU memory issues. Current deep neural network architectures generally put the skip connection around a stack of multiple layers^38,42,45^; they are primarily focused on classification problems for text or image classification. Here, we adapt the residual learning approach for vector-based regression problem, which is more pertinent to materials property prediction.

We introduce a novel approach of leveraging residual learning for each individual layer; referred to as individual residual learning (IRNet). Since each layer is non linear, being composed of a fully connected layer along with batch normalization^51^and ReLU^54^, we put a shortcut connection around each of them; the layer only learns the residual mapping from the input to the output, which makes it easy to train and converge. We find this results in better performance compared to existing approach of putting skip connection around a stack of multiple layers. IRNet architectures are designed for the prediction task of learning the formation enthalpy from a vector-based material representation composed of 145 composition-derived and 126 structure-derived attributes in the model input; trained using $\sim 436K$ samples from the Open Quantum Materials Database (OQMD)^2–4^; the 48-layer IRNet achieved a mean absolute error (MAE) of 0.038 eV/atom compared to an MAE of 0.072 eV/atom using Random Forest. A conference version of this work appeared in Jha et al.^55^; current article significantly expands on the conference paper with additional modeling experiments on more datasets, subsequent analysis of results and significant insights. We provide a detailed evaluation and analysis of IRNet on various publicly available materials datasets. We demonstrate the performance and robustness of IRNet against plain deep neural networks (without residual learning) and traditional machine learning algorithms for a wide variety of materials properties. We find that the use of individual residual learning in IRNet models can not only successfully alleviate the vanishing gradient problem and enable deeper learning, but also leads to significantly (up to 47%) better model accuracy as compared to traditional ML techniques for a given input materials representation, when big data is available. IRNet leverages a simple and intuitive approach of individual residual learning to build the deep neural networks without using any domain-dependent model engineering, which makes it attractive not only for the materials scientists, but also for other domain scientists in general to leverage it for their predictive modeling tasks on available big datasets.

## Results

### Datasets

We have used materials datasets from several sources to evaluate our models: Open Quantum Materials Database (OQMD)^4,56^, Automatic Flow of Materials Discovery Library (AFLOWLIB)^57^, Materials Project (MP)^58^, Joint Automated Repository for Various Integrated Simulations (JARVIS)^59–62^, and Matminer (an open source materials data mining toolkit)^63^. Dataset from OQMD is composed of 341,443 unique compositions (with each entry corresponding to the lowest energy crystal structure among all compounds with the same composition), with their DFT-computed materials properties comprising of formation enthalpy, band gap, energy per atom, and volume, as of May 2018. We also experiment using crystal structure as a part of model input for OQMD dataset; we refer to this dataset as OQMD-SC. OQMD-SC is composed of 435,582 unique compounds (unique combination of composition and crystal structure) with their DFT-computed formation enthalpy from the Open Quantum Database (OQMD)^4^; this is used for the design problem to find the model architectures^55^. Dataset from MP is composed of 83,989 inorganic compounds with a set of materials properties comprising of band gap, density, energy above hull, energy per atom, magnetization and volume, as of September 2018. AFLOWLIB dataset is composed of 234,299 compounds with materials properties comprising of formation energy, volume, density, energy per atom and band gap, as of January 2020. JARVIS dataset is downloaded from Matminer^63^ and is composed of 19,994 compounds with materials properties comprising of formation energy, band gap energy, bulk modulus and shear modulus, as of January 2020. We downloaded the Experimental Band-Gap dataset and Matbench Experimental Band-Gap dataset from Matminer^63^; they are composed of 6353 and 4603 inorganic semiconductor compounds, respectively, with the materials properties from^64^, as of January 2020. All evaluations use a hold out test set using a random train:test split of 9:1 (OQMD and MP datasets leverage the test set also as validation set during model training).

### Model architecture design

Since deeper neural network architectures have a larger capacity to learn a deeper hierarchy of abstract features, they are expected to have better performance, provided the architecture is designed well to address the performance degradation due to vanishing or exploding gradients problem^38,39,52^. Current deep neural networks architectures in computer science applications leverage hundreds of layers^37–45^; however, the existing DL works in materials science fail to leverage any deep architecture beyond $\sim 10$ layers^26,29,30^ (except 17-layer ElemNet^28^). Given the demonstrated potential and advantage of deeper architectures, and the increasing availability of big materials datasets containing hundreds of thousands of data points, it makes sense to leverage deeper architectures in materials science for better prediction performance. We introduce a novel approach of using residual learning at each layer for fully connected deep neural networks to solve the issue of performance degradation due to vanishing and/or exploding gradients; we refer to this approach as individual residual learning (IRNet). IRNet takes a vector-based material representation as model input and is composed of fully connected layers. Since fully connected layers have a huge number of parameters, we leverage the residual learning approach based on He et al.^38^ to limit the number of additional model parameters so that they could fit in GPU memory. IRNet learns to predict materials properties as the model output, which are continuous values (hence, a regression task).

IRNet architectures are designed on the prediction task of learning the formation enthalpy from a vector based materials representation composed of 126 structure-derived attributes (using Voronoi tesselation from Ward et al.^65^) and 145 composition-derived physical attributes using OQMD-SC; OQMD-SC is composed of 435,582 samples; we split the dataset randomly into 9:1 training and test (validation) splits. The deep neural network architectures are composed of fully connected layers; each fully connected layer being followed by batch normalization^51^, and ReLU^54^ as the activation function. IRNet uses a novel approach of shortcut connection for residual learning around each fully connected layer for better gradients flow. To demonstrate the impact of our novel approach of residual learning, we also design a plain network and a stacked residual network (SRNet). The plain networks do not leverage any shortcut connection for residual learning; SRNets place shortcut connection around a stack of layers with exactly same configuration, similar to the existing approach in computer science applications^38,42,45^. The model architectures for all the models used in this work are provided in the Methods section.

**Figure 1:**
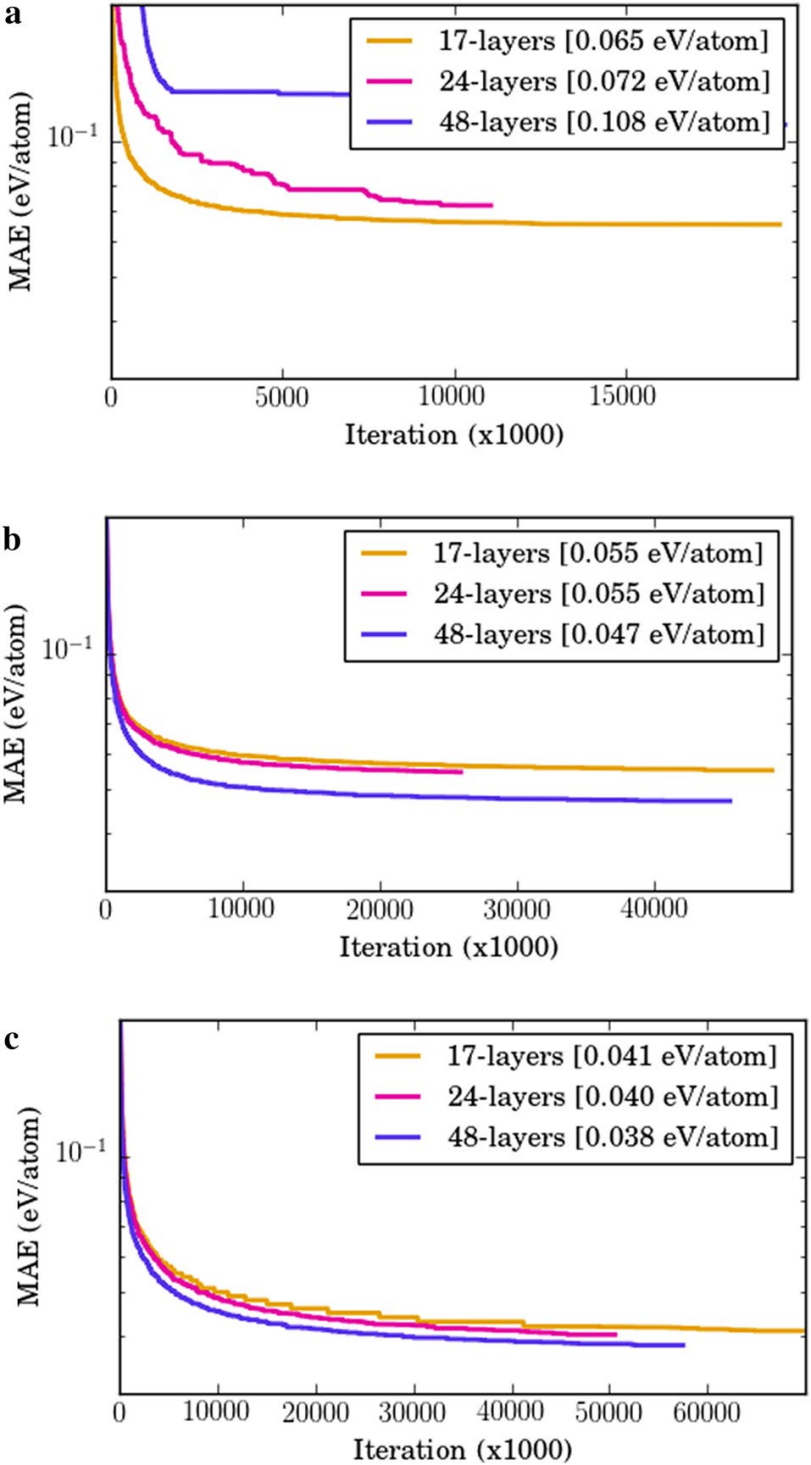
Impact of residual learning for the design problem. Design problem involves predicting formation enthalpy from vector-based materials representation composed of 126 structure-derived and 145 composition-derived physical attributes on the OQMD-SC. They are trained using 9:1 random train:test splits (test set is same as validation set). Plain Network do not have shortcut connections; stacked residual network (SRNet) places shortcut connection after stacks of multiple layers; individual residual network (IRNet) leverage individual residual learning around each layer. The three subplots shows the validation error curves during training for each network; x-axis represents the training iteration (x1000) and y-axis represents the MAE. The models are implemented using TensorFlow and trained using Adam optimizer with a mini batch size of 32 and a learning rate of 1e-4 and a patience of 400 epochs (training stops if the validation error does not improve for last 400 epochs).

As we can observe in Fig. 1, the plain network performance degrades with increase in depth; the 17-layer performs better than 24-layer and the 24-layer performs better than the 48-layer. The performance of a plain network deteriorates with the increase in depth of the architecture due to vanishing and/or exploding gradient issue, even in the presence of batch normalization^51^; the MAE increases from 0.065 eV/atom for 17-layer to 0.072 eV/atom for 24-layer and even worse 0.108 eV/atom in the case of 48-layer plain network. The use of residual learning solves this issue of performance degradation with increase in architecture depth as we can observe in the case of SRNet and IRNet; the use of shortcut connections around the non linear fully connected layers (with batch normalization and ReLU activation) helps in gradients flow during backpropagation even for very deep neural network architectures. When leveraging residual learning, we observe significant benefit from increasing depth in both cases. The benefit of leveraging deeper architecture becomes clear when we increase the depth to 48-layer in both cases. The MAE values decreases to 0.047 eV/atom and 0.038 eV/atom for 48-layer compared to 0.055 eV/atom and 0.041 eV/atom for 17-layer, for SRNet and IRNet, respectively. For the given design problem, we observe that IRNet leads to better convergence during training and significantly outperform the plain network and the SRNets (which are based on existing approach of residual learning). We also trained traditional ML algorithms such as Linear Regression, SGDRegression, ElasticNet, AdaBoost, Ridge, RBFSVM, DecisionTree, Bagging and Random Forest on this prediction task. While IRNet (48-layer) achieved an MAE of 0.0382 eV/atom on the design problem task; the best plain network (17-layer) achieved an MAE of 0.0653 eV/atom, and Random Forest (best traditional ML algorithm for the given prediction task^13^) achieved an MAE of 0.072 eV/atom. IRNet helped in significantly reducing the prediction error by $\sim 47\%$ compared to traditional ML. This illustrates the benefit of leveraging our novel approach of individual residual learning (IRNet) compared to traditional ML, plain networks, and existing residual learning networks (SRNet) for the design task.

### Composition as model input

Next, we demonstrate the significance for residual learning on the prediction modeling tasks of “materials properties given composition”. We train IRNets of different depths: 17-layer and 48-layer, for predicting materials properties from vector-based material representation composed of 145 composition-derived physical attributes (computed using MagPie^13^) as model input. To illustrate the impact of residual learning, we also train 17-layer plain networks. We compare the performance of DL models against traditional ML algorithms: Linear Regression, Ridge, Decision Tree, ExtraTrees, Bagging, AdaBoost, ElasticNet, SGD Regression, Random Forest and Support Vector Machines; we carry out extensive grid search to tune their hyperparameters for each of these algorithms. We observe in Table 1 that the 17-layer IRNet always outperforms the 17-layer plain network and the traditional ML algorithms. The performance of 17-layer plain network is better than the traditional machine learning approach in general, but significantly worse than 17-layer IRNet. Since the plain network does not have any shortcut connection for residual learning, they are not immune from the performance degradation issue due to vanishing and/or exploding gradients. IRNet significantly benefits from the use of shortcut connections for individual residual learning, which helps with smooth gradient flow during backpropagation. For the two datasets with more than 100K samples: OQMD and AFLOWLIB, we find that the 48-layer IRNets outperform the 17-layer IRNets; the difference in performance is more significant for OQMD than for AFLOWLIB. For MP with data size < 100K, we find that 17-layer IRNets perform better than 48-layer IRNets; this may be because the 48-layer IRNets can overfit to the training data due to the large number of parameters when data size is not very big. From this analysis, we conclude that the depth of neural network architectures should depend on the size of the available dataset, with deeper residual learning architectures providing better performance when bigger data are available. IRNet clearly outperforms the traditional machine learning algorithms and plain networks for almost all materials properties in the four datasets used in this performance evaluation analysis. This clearly illustrates the benefit of leveraging deeper architectures with individual residual learning for the given prediction task of “materials properties given composition” in the presence of big data.

**Table 1:** Performance benchmarking for the prediction task of “materials property given composition”.

Dataset	Property	Size	Best of 10 ML	17-layer plain network	17-layer IRNet	48-layer IRNet
OQMD	Formation enthalpy (eV/atom)	341,443	0.077	0.072	0.054	**0.048**
	Bandgap (eV)	341,443	**0.047**	0.052	0.051	**0.047**
	Volume_pa ($\hbox {A}^3$)	341,443	0.473	0.483	0.415	**0.394**
AFLOWLIB	Formation enthalpy (eV/atom)	234,299	0.067	0.076	**0.059**	**0.059**
	Volume_pa ($\hbox {A}^3$)	234,299	0.742	0.749	0.668	**0.663**
	Density ($\hbox {grams/cm}^3$)	234,299	0.235	0.232	0.209	**0.201**
MP	Formation energy_per_atom (eV)	89,339	0.136	0.153	0.132	**0.131**
	Bandgap (eV)	83,989	0.479	0.396	**0.363**	0.364
	Density ($\hbox {grams/cm}^3$)	83,989	0.505	0.401	**0.348**	0.386
	Total_magnetization ($\mu _B$)	83,989	3.232	3.090	**3.005**	–
	Volume ($\hbox {A}^3$/lattice)	83,989	225.671	219.439	**215.037**	–
JARVIS	Formation enthalpy (eV/atom)	19,994	0.113	0.150	**0.108**	0.114
	Bandgap (eV)	17,929	0.375	0.363	**0.311**	–

The number in bold font represents the best model performance for a given combination of dataset, materials property and model input (each row).

### Structure as model input

Next, we illustrate the versatility of leveraging deeper architectures with residual learning by building models with additional structure-derived attributes in the vector-based materials representation for model input. We train IRNets, plain networks and traditional ML algorithms similar to previous analysis, but use different combinations of model inputs with varying length. For model input, we use 126 structure-derived attributes (structure) using Voronoi tesselation^65^, 145 composition-derived physical attributes (comp) (computed using MagPie^13^), and 86 elemental fractions (EF)^28^. Table 2 demonstrates the performance of IRNet models using different types of materials representations in the model input for datasets (with required structure information for computing attributes using Voronoi tesselation). Generally, we observe that models based on only using materials composition perform better than models based on only using materials structure, for all types of machine learning models for all datasets used in our study. While structure by itself does not work well, it significantly improves the performance of models if used along with composition. For 17-layer IRNet, we find that the improvement in performance by adding structure to the model input increases with increase in dataset size. For instance, the performance of 17-layer IRNet to predict formation energy, improves from an MAE of 0.054 eV/atom to 0.041 eV/atom (around 24%) for OQMD compared to improvement in MAE from 0.108 eV/atom to 0.097 eV/atom (around 10%) for JARVIS, when adding structure with composition as input to the model. For JARVIS, we find that plain network performs worse than the traditional ML algorithms; this is because these datasets are comparatively smaller in size (containing $\sim 20-45K$), However, we can observe that IRNet performs better than both plain network and traditional ML algorithms. The individual residual learning approach used in IRNet appears to significantly help them in capturing the materials properties from the given materials representations, which the plain network fails to learn well. An interesting observation from Table 2 is that the increase in the number of attributes in the model input results in better performance for all types of models: traditional ML algorithms, plain network as well as IRNet; using composition-derived attributes along with structure-derived attributes is better than using the structure-derived attributes alone; adding elemental fractions to the model input also slightly improves the performance. This illustrates the versatility of leveraging individual residual learning for enabling deeper model architectures for the general prediction modeling task of materials property given any type of vector-based materials representation in the presence of big data.

**Table 2:** Performance benchmarking for the prediction task of “materials property given structure”.

Dataset	Property	Model input	Size	Best of 10 ML	17-layer plain network	17-layer IRNet
JARVIS	Formation enthalpy (eV/atom)	Structure	25,405	0.125	0.153	**0.114**
		Structure+Comp	25,405	0.107	0.129	**0.097**
		Structure+Comp+EF	25,405	0.107	0.125	**0.096**
	Bandgap (eV)	Structure	22,952	0.338	0.400	**0.337**
		Structure+Comp	22,952	0.284	0.336	**0.280**
		Structure+Comp+EF	22,952	0.280	0.335	**0.276**
OQMD	Formation enthalpy (eV/atom)	Structure	435,582	0.106	0.102	**0.072**
		Structure+Comp	435,582	0.072	0.065	**0.041**
	Bandgap (eV)	Structure	435,293	0.056	0.075	**0.071**
		Structure+Comp	435,293	0.045	0.046	**0.042**
	Volume_pa ($\hbox {A}^3$)	Structure	435,625	1.032	1.806	**1.684**
		Structure+Comp	435,625	0.451	0.385	**0.287**

The number in bold font represents the best model performance for a given combination of dataset, materials property and model input (each row).

### Performance on smaller datasets

**Figure 2:**
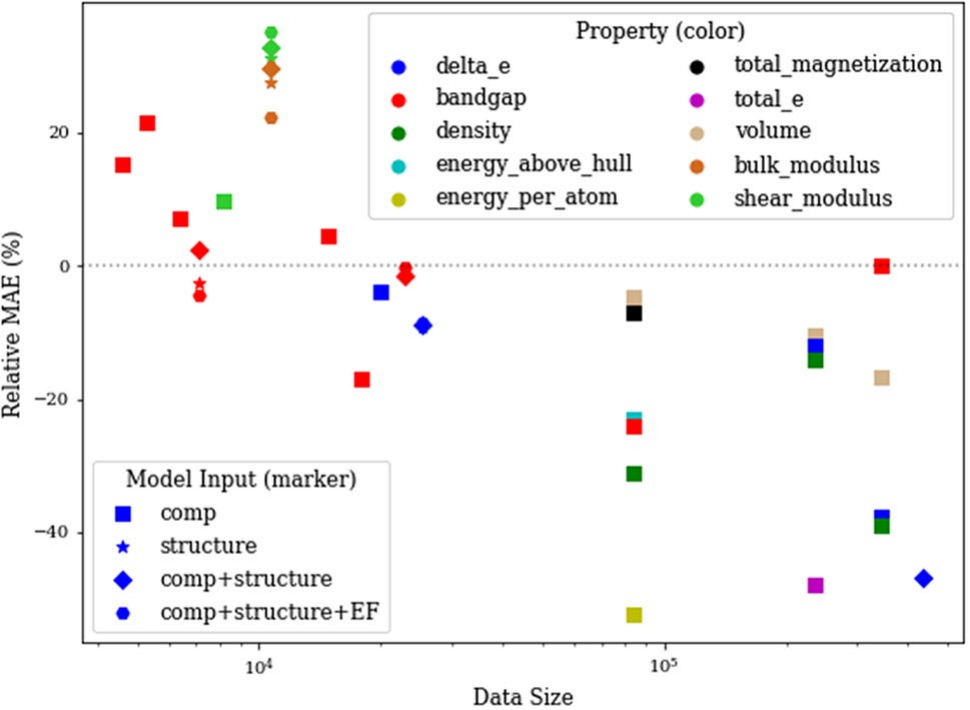
Impact of data size on the performance of IRNet. X-axis shows the dataset size on log scale, and Y-axis shows the percentage change in MAE of IRNet w.r.t. the best traditional ML model (calculated as $(MAE_{IRNet}/MAE_{BestML}-1)\times 100\%$). We plot the performance of IRNet and best ML model from all the experiment in our study. Note that the reported MAE are on a hold-out test set using a 9:1 random train:test split (same test set is used as validation for OQMD and MP).

In our analysis, we generally observe the benefit of leveraging individual residual learning to enable deeper model architectures which tend to perform better than the plain networks and traditional ML models if big data is available. Here, we investigate the limitations of deeper model architectures (IRNet) by evaluating their performance against traditional ML algorithms on datasets $\sim 10K$ in size in Table 3. For these smaller datasets, we also designed a 10-layer IRNet so that the model does not overfit to the training data. As we can observe, 10-layer IRNet generally performs comparable to 17-layer IRNet on these prediction tasks since the datasets are small. We observe that traditional ML algorithms generally perform better for all types of materials representations in the model input. Similar to previous analysis, they benefit from combining structure-derived and composition-derived attributes in the model input in general for traditional machine learning models but not necessarily for IRNet. For instance, IRNet performs better while using composition-derived attributes alone than combining them with structure-derived attributes for two out of three materials properties for JARVIS, while they perform best when we use all three types of materials representation together. Furthermore, we illustrate the benefit of using IRNet and impact of big data in Fig. 2. We can observe that IRNet consistently outperforms traditional machine learning algorithms once the dataset size is big enough (exceeds $\sim 15K$ in size). This observation is independent of material representation used in the model input and the materials property in the model output. We hope this will motivate materials scientists in leveraging individual residual learning to build their deep neural network architectures when large datasets are available.

**Table 3:** Performance of IRNet on smaller datasets.

Dataset	Property	Model Input	Size	Best of 10 ML	10-layer IRNet	17-layer IRNet
AFLOWLIB	Bandgap (eV)	Comp	14,751	**0.112**	–	0.124
JARVIS	Bulk Modulus (GPa)	Comp	8205	**12.204**	12.974	13.409
		Structure	10,707	**14.687**	17.213	17.978
		Structure+Comp	10,707	**10.624**	14.381	14.121
		Structure+Comp+EF	10,707	**10.530**	12.651	13.429
	Shear modulus (GPa)	Comp	8205	**10.488**	11.804	11.513
		Structure	10,707	**11.138**	13.689	15.067
		Structure+Comp	10,707	**9.406**	12.237	12.350
		Structure+Comp+EF	10,707	**9.370**	11.640	12.158
	Bandgap (eV)	Comp	5299	**0.572**	0.628	0.695
		Structure	7136	**0.566**	0.613	0.541
		Structure+Comp	7136	**0.505**	**0.505**	0.517
		Structure+Comp+EF	7136	0.499	0.544	**0.485**
Exp Bandgap	Bandgap (eV)	Comp	6353	**0.307**	0.333	0.328
Matbench Exp Bandgap	Bandgap (eV)	Comp	4603	**0.364**	0.461	0.419

The number in bold font represents the best model performance for a given combination of dataset, materials property and model input (each row).

### Prediction error analysis

**Figure 3:**
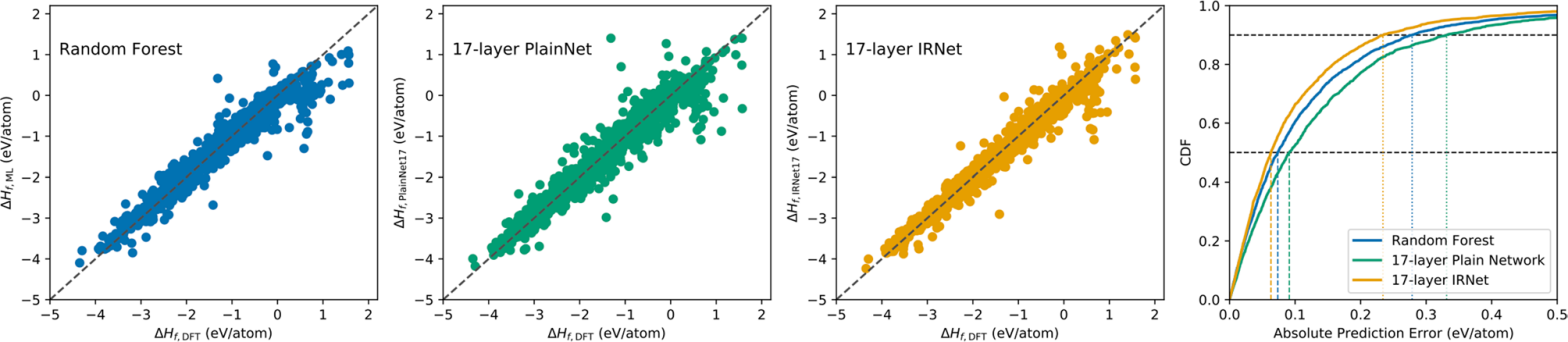
Prediction error analysis for prediction formation enthalpy in JARVIS dataset using different models. We use the 145 physical attributes derived from material composition as the model inputs. We benchmark against plain network and several traditional ML models such as Linear Regression, SGDRegression, ElasticNet, AdaBoost, Ridge, RBFSVM, DecisionTree, ExtraTrees, Bagging and Random Forest, with exhaustive grid search for hyperparameters; Random Forest performed best among traditional ML algorithms. We use the prediction errors on the hold-out test set using a random 9:1 train:test split. The first three subplots represent the prediction errors using three models: Random Forest, 17-layer Plain Network and 17-layer IRNet; the last subplot contains the cumulative distribution function (CDF) of the prediction errors using the three models.

Next, we analyze the prediction error distribution for different combinations of model input, model type and output property in our analysis. Fig. 3 illustrates the prediction error distribution for formation enthalpy in JARVIS dataset using different models with the 145 composition-derived physical attributes. Since the JARVIS dataset is comparatively smaller ($\sim 20K$), we observe that the plain network performs worse than Random Forest. The IRNet model benefits from leveraging individual residual learning and outperforms Random Forest. Although the scatter plot of IRNet is more similar to the plain network, we can observe that the prediction moves closer to diagonal. Scatter plots illustrate that all the three models have outliers, with outliers in the case of IRNet being relatively closer to the diagonal. The difference in prediction error distributions becomes more evident from the CDF (cumulative distributive function) curves for the three models. The 90th percentile absolute prediction error for IRNet is significantly lower than Random Forest and plain network; this illustrates the robustness of IRNet against Random Forest and plain network. We find similar trends in the scatter plot and CDF of prediction errors for other properties in other datasets in our study. Our observations demonstrate that one can improve the performance and robustness of their DL model by leveraging individual residual connections in the presence of large data.

**Figure 4:**
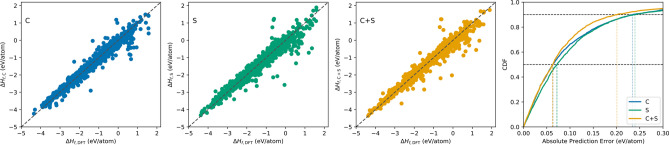
Prediction error analysis for formation enthalpy in JARVIS using different model input for 17-layer IRNet. We use the prediction errors on the hold-out test set using a random 9:1 train:test split. The first three subplots represent the prediction errors on the hold-out test set using three different model inputs for IRNet: C (composition-derived 145 physical attributes), S (126 structure-derived attributes using Voronoi tesselation) and C+S (145 physical attributes+126 structural attributes); the last subplot contains the CDF of the prediction errors for the three model inputs for IRNet.

Furthermore, we investigated the impact of including different types of material representation in the model input by plotting their prediction error scatter plots and CDFs. Figure 4 illustrates prediction error distributions for predicting formation enthalpy in JARVIS using different types of material representations in the model input: C (composition-derived 145 physical attributes^13^) and S (126 structure-derived attributes using Voronoi tessellation^65^). From the scatter plots, we observe that leveraging composition-derived attributes as model inputs provide better predictions compared to structure-derived attributes; structure-derived attributes result in more scattered predictions with expanded distribution and more outliers compared to composition-derived attributes. This is also clear from the MAE values reported in Tables 1 and 2. Combining the composition-derived and structure-derived attributes clearly leads to better prediction performance. The third scatter plot illustrates that predictions moves more closer to diagonal, resulting in better performance. This observation becomes more distinct if we analyze their respective CDF plots. We observe that leveraging both types of material representation in the model input moves the CDF towards left; this is especially true for the predictions with absolute error higher than 60th percentile. We observe similar trend for other models for predicting other properties in our analysis. These prediction error analyses demonstrate the significance of leveraging residual learning with inclusion of all available material representations in the model input for better prediction performance.

## Discussion

We presented a novel approach of leveraging individual residual network to enable deeper learning on big data for materials property prediction. To illustrate the benefit of leveraging the proposed approach, we built a general deep learning framework composed of deep neural network architectures of varying depth (10-layer, 17-layer, 24-layer, 48-layer), referred as IRNet. To compare the performance of IRNet, we built plain network with no residual learning, and stacked residual network (SRNet) based on current approach of residual learning. The presented IRNet architectures were designed (optimized) for the task of predicting formation enthalpy using a vector-based material representation composed of 145 composition-derived and 126 structure-derived attributes in the model input. On the design problem, IRNet leveraging the proposed design approach significantly outperformed the traditional ML algorithms, plain network and SRNet. We demonstrated the efficacy of the proposed approach by evaluating and comparing these DL model architectures against plain network architecture and several traditional ML algorithms on a variety of materials properties in the available materials datasets. Furthermore, we demonstrated that the presented DL model architectures leveraging the proposed approach are versatile in their vector-based model input by evaluating prediction models for different materials properties using different combination of vector-based material representations: composition-derived 145 physical attributes and/or 126 structure-derived attributes with(out) 86 raw elemental fractions. Our analysis demonstrates that prediction models generally benefit from leveraging all available material representations in the model input. The availability of big data appears to benefit deep neural network architectures in achieving better prediction performance as expected; deeper architectures result in better prediction models since they have better capability to capture the mapping between the given input material representation and the output property. The training time of deep neural network architectures depends on the given prediction task (model inputs and model output), the size of training dataset, and the depth of the neural networks (number of model parameters); the use of individual residual learning in IRNet does not have any significance increase in the training or inference time. For instance, the training time for IRNet can range from a few hours for small datasets such as JARVIS, to a couple for days for big datasets such as OQMD on GPUs (such as Tesla V100 used in our study) for the prediction task of formation energy given materials composition and structure as model input (compared to traditional ML algorithms taking only upto a couple of hours, but they poorly scale with increase in training dataset size)^28^; however, this is a one time cost. Nevertheless, deep neural networks like IRNets can be significantly faster (upto an order of two) in making predictions while running on GPUs compared to traditional ML models such as Random Forest, which are typically run on CPUs. Hence, deep neural networks can make it feasible to screen millions of hypothetical potential materials candidates in a few hours, making them ideal for applications in materials discovery and design^28^. Since the presented approach of leveraging individual residual learning in IRNet does not depend on any particular material representation/embedding as model input, we expect that the presented approach of leveraging individual residual learning to enable deeper learning can be used to improve the performance of other DL works leveraging other types of materials representations in materials science and other scientific domains; we plan to explore them in future. The presented technique of individual residual learning is conceptually simple and elegant to implement and build upon; the IRNet framework code is publicly available at https://github.com/dipendra009/IRNet.

## Methods

### Model architectures

**Figure 5:**
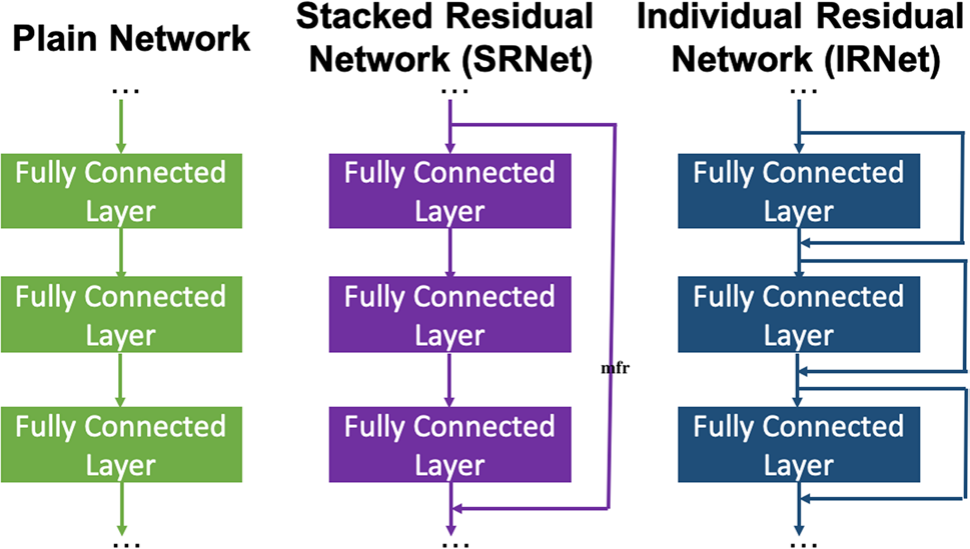
Design approach for IRNet. Plain Network is composed of sequence of fully connected layer, where each layer is composed of a dense layer followed by batch normalization^51^ and ReLU^54^. Existing approach of residual learning places shortcut connection around each stack of multiple such layers where all the layers within each stack have same configuration (SRNet). The presented approach of individual residual network (IRNet) places shortcut connection around each layer which makes it easy for the model to learn the mapping of output materials property from the material composition and/or structure in the model input.

The design approach for IRNet is illustrated in Fig. 5. The model architecture is formed by putting together a series of stacks, each composed of one or more sequences of three basic components with the same configuration. Since the input is a numerical vector, the model uses a fully connected layer as the initial layer in each sequence. Next, to reduce the internal covariance drift for proper gradient flow during back propagation for faster convergence, a batch normalization layer is placed after the fully connected layer^51^. Finally, ReLU^54^ is used as the activation function after the batch normalization. The simplest instantiation of this architecture adds no shortcut connections and thus learns simply the approximate mapping from input to output. We refer to this network as a *plain network*. We use stacks of consecutive layers with the same configuration, with the first stack composed of four sequence of layers and the final stack of two sequences. He et al.^38^ introduced the idea of using shortcut connections after a stack composed of multiple convolutional layers. In our case, the stacks are composed of up to four sequences, with each sequence containing a fully connected layer, a batch normalization, and ReLU. We place a shortcut connection after every sequence, so that each sequence needs only to learn the residual mapping between its input and output. This innovation has the effect of making the regression learning task easier. As each “stack” now comprises a single sequence, shortcut connections across each sequence provide a smooth flow of gradients between layers. We refer to such a deep regression network with individual residual learning capability as an *individual residual network* (IRNet). The detailed architectures for all the networks with different depths are provided in Table 4 (the notation [...] represents a stack of model components, comprising of a single sequence (FC: fully connected layer, BN: batch normalization, Re: ReLU activation function) in the case of IRNet; each such stack is followedby a shortcut connection); the implementation of all the models used in this work is publicly available at https://github.com/dipendra009/IRNet.

**Table 4:** Detailed model architecture configurations for different depths of network architecture.

Output	10-layer IRNet	17-layer IRNet	48-layer IRNet
1024	[FC1024-BN-Re] x 2	[FC1024-BN-Re] x 4	[FC1024-BN-Re] x 8
512	[FC512-BN-Re] x 2	[FC512-BN-Re] x 3	[FC512-BN-Re] x 8
256	[FC256-BN-Re] x 2	[FC256-BN-Re] x 3	[FC1024-BN-Re] x 8
128	[FC128-BN-Re]	[FC128-BN-Re] x 3	[FC128-BN-Re] x 8
64	[FC64-BN-Re]	[FC64-BN-Re] x 2	[FC64-BN-Re] x 8
32	[FC32-BN-Re]	[FC32-BN-Re]	[FC32-BN-Re] x 4
16			[FC16-BN-Re] x 3
1	FC1		

### Network and ML settings

We implement the deep learning models with Python and TensorFlow 1^66^. We found the best hyperparameters to be Adam^67^ as the optimizer with a mini batch size of 32, learning rate of 0.0001, mean absolute error as loss function, and ReLU as activation function, with the final regression layer having no activation function. Rather than training the model for a specific number of epochs, we used early stopping with a patience of 200 epochs (except for design problem which used a patience of 400 epochs), meaning that we stopped training when the performance did not improve in 400 epochs. For traditional ML models, we used Scikit-learn^68^ implementations and employed mean absolute error (MAE) as loss function and error metric. We carried out extensive hyperparameter grid search for all the traditional ML methods used in this work.

## Data Availability

All the datasets used in this paper are publicly available from their corresponding websites-OQMD (http://oqmd.org), AFLOWLIB (http://aflowlib.org), Materials Project (https://materialsproject.org), JARVIS (https://jarvis.nist.gov), and using Matminer (https://hackingmaterials.lbl.gov/matminer/).
